# Wills Hospital for the Diseases of the Eye

**Published:** 1854-09

**Authors:** 


### MEDICAL EXAMINER.

NEW SERIES.—NO. CXVII.—SEPTEMBER, 1854.

### ORIGINAL COMMUNICATIONS.

*Wills Hospital for the Diseases of the Eye.* Service of Dr. LITTELL. *Abstract of the Quarterly Report.*

On the first of April, there were in the Hospital, thirty patients, of whom 21 were males and 9 females. Seventy others —46 males and 24 females—were admitted during the Quarter. Total 100. Of the admissions, 26 were in April, 26 in May, and 18 in June.

Sixty-two persons—42 males and 20 females—were discharged in the same period. Of these, fifty-nine were cured, two relieved and one eloped. Leaving in the Hospital, June 30th, 1854, thirty-eight patients, of whom 24 were males and 14 females.

There were 392 new out-patients reported by the resident physician, Dr. Homan; but as during the first few weeks of his incumbency, those only were recorded who presented themselves on the regular prescribing days, the number may very safely be estimated at 400. Of those officially reported, 78 applied in April, 155 in May and 159 in June. The number of in- and out-door patients, under treatment during the Quarter, was, therefore, 500; exclusive of those who still attended from the preceding term. The rule lately adopted of sending the out-patients elsewhere for their medicines, appears to answer very well; people setting a higher value on that for which they pay something.

The majority of the cases consisted of chronic conjunctivitis, alone or complicated with granular lids and vascular cornea, phlyctenular ophthalmia, catarrho-rheumatic ophthalmia, catarrhal and purulent ophthalmia, iritis, ulcers of the cornea, cataract, amaurosis, &c. &c.; and, in general, presented no features worthy of particular commemoration. A few may be selected for the practical lessons which they inculcate.

A tall, thin, pale and delicate girl was admitted with violent internal inflammation of the right eye; there was a large effusion into the anterior chamber of a puro-lymphatic fluid, contraction of the pupil, intolerance of light, lachrymation, &c. The part more particularly involved appeared to be the lining membrane of the cornea, and the severity of the local symptoms seemed to call for depletory measures; which, on the other hand, were forbidden by the state of the pulse, and the general feeble health of the patient. The atony of the extreme vessels is proportionate to the general depression, and inflammation tending to rapid disorganization sometimes occurring under such circumstances, serious consequences would ensue from any thing that might still further impair the vital powers. The neuralgic pain, frequently a prominent subject of complaint in these anemic conditions, is a wise exertion of the vis medicatrix, designed to preserve the integrity of the part, by making it the centre of increased action and determination. Even in cases of less general depravation, where the intensity of the symptoms imperiously demands local depletion, it will often be necessary to support simultaneously the nervous energy; and thereby restore the impaired tonicity of the capillaries. A combined tonic and alterative treatment—half a grain of calomel, and a grain and a half of quinine, three times a day—was, therefore, adopted in the present instance; and, with full diet and other invigorating measures, steadily pursued for six weeks; at the end of which time the inflammation began to abate, the exudation to lessen, and in a little while thereafter, all traces of disease had disappeared, leaving the cornea clear and the iris free.

A case of ulceration of the cornea following small pox, may also be mentioned, in illustration of the treatment required in inflammation supervening on exhausting diseases. In this form of secondary variolous ophthalmia, often occurring a week or more after the decline of the eruption, the cornea becomes opaque in its central portion, but this opacity, which has been erroneously denominated pustular, is more properly the actual death of the part from inadequate nutrition. It certainly presents none of the characteristics of pustule, contains no purulent matter, and has no specific property. When separation of the slough commences through the ulcerative process, inflammation is set up to develope those actions which are necessary for the reparation of the mischief; and, provided it do not transcend the required degree, new structure is formed by the deposition of lymph. The patient, in the present instance, had recovered from the confluent variety of the complaint, but the cornea had become partially opaque; and at the time of his admission, ulceration had already begun, and the eye was much inflamed, with lachrymation, intolerance of light, &c. The gravity of the constitutional malady had been such as to induce great debility of the system, atony of the extreme vessels, and an altered condition of the fluids, ending in the destruction of a remote and imperfectly organized portion of the body; and it was evident, therefore, that the treatment of the case should be both nutritive and tonic. A generous diet was accordingly directed, two grains of quinine, and half an ounce of the ol. morrhuæ, with a small proportion of the spirits of turpentine, were prescribed three times a day; opium was given to allay pain and procure sleep, and an occasional application was made of a weak solution of the nitrate of silver. The result of the case was in the highest degree satisfactory. The ulceration beginning at a point of the circumference of the opacity, gradually extended until the whole was removed; but, seated in the conjunctiva corneæ, it happily involved, in very slight degree, the proper texture of that membrane, and the part finally healed with little diminution of its transparency.

A chronic case of iritis, insidious in its progress, and affecting chiefly the posterior surface of the iris, was unusually intractable. The patient was a young woman of ordinary health and vigor, and the disease had already existed three months. She was bled moderately, and leeches were applied to the temple with relief of the pain; after which, a grain of calomel with the sixth of a grain of opium was prescribed, and continued three times a day, for the space of two or three weeks, when a grain of quinine was substituted for the opium. The treatment was subsequently varied by the prescription of the sixteenth of a grain of the bichloride of mercury with a drachm of the compound tincture of cinchona, and this again was in turn relinquished for the solution of the arsenite of potash. All the remedies were productive of good effect; but the improvement was apparently greater under the two last; probably from the impulse towards recovery already given by the former.

Another case of uveitis presenting for a considerable period no objective symptoms, and in which at first the only abnormal deviation was near-sightedness and dimness of vision, such as might be produced by an increased secretion of the aqueous humor, was treated successfully after the same manner. Entire rest of the organ was enjoined, a few leeches were applied to the temple, the dilatation of the pupil maintained by means of atropia, and the mild and corrosive chlorides of mercury successively administered, in combination with quinine and the tincture of bark.

To the eye of a patient previously to her application at the hospital the acetate of lead had been applied as a lotion during the existence of an ulcer immediately opposite the pupil; and the consequence was a dense opacity of the cornea which baffled all attempts at removal. This accident, which is not of unfrequent occurrence, is very liable to happen whenever ulceration is present, and would probably always do so, but that in some individuals the lachrymal secretion is so slightly impregnated with saline ingredients as to produce little or no precipitation. Opacity from this cause, when slight and of recent origin, will, it is said, sometimes disappear under the persevering use of a weak collyrium of acetic acid, but on trial at the Hospital this has not been confirmed; the deposit being a muriate and becoming incorporated more or less intimately with the texture of the part, is, moreover, not likely to be removed by such means.

In phlyctenular ophthalmia, the primary indication is the correction of any functional derangement, more especially of the digestive system. This being accomplished by the occasional exhibition of calomel or hydrargyrum cum creta, with rhubarb, the cure is completed by the sulphate of quinine, combined, if there be much photophobia, with belladonna, or the solution of morphia; and the employment, as an external application, of a weak collyrium of the nitrate of silver. The term "scrofulous," sometimes applied to this complaint, is an unfortunate appellation, inasmuch as by the false pathology which it involves, it is liable to mislead the practitioner, by inducing him to seek for remedies of a peculiar alterative character, for the relief of what is merely an inflammation of weak action occurring in a lymphatic temperament.

The treatment of gonorrhœal ophthalmia generally errs on the side of activity, and the inflammation, from the impairment of the recuperative energies thereby induced, is often rendered more destructive than it would otherwise be. Moderate depletion, general or local, as circumstances may require, opiates in full doses at night, the solution of the nitrate of silver in the strength of from four to ten or twenty grains, and the radiated incision of the chemosed conjunctiva when the cornea is theatened, are the appropriate remedies. Persons frequently apply at the Hospital with this membrane in a sloughing condition, from a treatment either too active or ill-directed. This is especially liable to happen in the purulent ophthalmia of infants, owing to an erroneous belief that it is caused by the impression of light, and is therefore a trivial affection requiring little attention; whereas, in every instance it arises from contact with the vaginal secretion of the mother, and is often a formidable malady.

A young man was admitted with gonorrhœal ophthalmia, which began a week before, and affected in the first instance the left eye only. Every therapeutic arm had been vigorously employed in its reduction. He had been bled largely, cupped, purged, dieted, and mercurialized. At the time of his admission, there was sloughing of the cornea at its inner and lower portion, escape of the aqueous humor, and collapse of the iris. The disease had attacked the right eye with almost equal severity a day or two previously, and there was in both a considerable degree of chemosis, with a profuse purulent discharge. His strength had been already greatly reduced by the active treatment to which he had been subjected; and he was, moreover, much exhausted by pain and want of sleep. In connection with the general debility the capillaries of the part were in a state of extreme atony; a condition which, though often overlooked, occurs also in the latter stages of bronchitis and other affections of the mucous tissue, and requires in both cases a corresponding treatment. He was placed on a full diet, with half an ounce of cod-liver oil and a few drops of the spirits of turpentine after every meal; two grains of quinine were given every four hours during the day, and a grain and a half of opium with three grains of hyosciamus at bed-time. A bottle of porter daily was subsequently directed. As local applications, a solution of the nitrate of silver—four grains to the ounce—was dropped into the eye once a day, the purulent secretion frequently washed away with a weak collyrium of the bi-chloride of mercury—two grains to the pint—and the dilatation of the pupil maintained, as far as practicable, by atropia. The beneficial influence of these measures was great and immediate; the pain was relieved, the inflammation rapidly subsided, and the patient was discharged with his right eye uninjured, and vision in the left, owing happily to the partial disengagement of the iris, very little impaired.

By far the most frequent of the morbid alterations treated at the Hospital is an hypertrophied condition of the lining membrane of the lids, with its sequelæ, vascular cornea, opacity, ulceration, &c. Cases of this nature require, for the most part, an invigorating and nutritive treatment. The improvement of the general health, indeed, is often the primary consideration. All those measures, therefore, which conduce to this end are sedulously called into requisition. The stimulus of hope, a generous diet, exercise in the open air, the shower bath when the season will permit, adequate clothing, the ol. morrhuæ, quinine, the chalybeates, the compound infusion of gentian, the preparations of iodine, the hydrargyrum cum creta with aloes and hyosciamus; and generally, such other remedies as are adapted to invigorate the system, regulate the bowels, and correct functional derangement, may be severally or jointly prescribed with advantage under such circumstances. Concurrently with these means, and more particularly, after the health has begun to improve under their use, local applications are demanded; and of these the most effectual are the nitrate of silver and the sulphate of copper. The first is employed in solution, of a strength varying from four to thirty grains to the ounce, and is applied by means of a camel-hair pencil to the everted lid, daily, every alternate day, or twice a week, according to the circumstances of the case and the strength of the solution; time being allowed for the subsidence of the vascular excitement thus temporarily increased, and also for the production of the expected curative impression. The stronger solutions are seldom used unless the granulations are large, or the case so chronic that the membrane has already become disorganized, and then only after intervals of three or four days. In general, care should be taken not to employ measures of such activity as occasion much reaction, or would be likely to destroy or permanently injure the texture of the part. The morbid degeneration of months or years cannot be soon repaired; patience and perseverance are required both of the patient and the physician; and to no complaint is the maxim, festina lente, more appropriate. The sulphate of copper, lightly applied in substance, is a remedy of nearly equal value with the nitrate of silver; and, as being more tonic in its impression, is perhaps even better adapted to cases in which the granulations are soft and small. Other substances are used with the same view, as crystals of the sulphate of alumine and the borate of soda, and also various astringent solutions; but the two salts above mentioned, are, as a general rule, preferable to all others at present known. Scarifications though perhaps, sometimes useful when the membrane is the seat of much vascular congestion, should not be generally or frequently employed; but the excision of the granulations by the scissors, or the ablation of the diseased tissue by the knife, as is still practised by some surgeons, cannot be too severely reprehended. Such modes of treatment are not only rude and unscientific, but they indefinitely protract the cure, and increase the tendency to incurvation of the cartilage which already exists, and is itself an obstacle to recovery. In some cases where the lid, owing to this contraction, appears to embrace the globe too closely, the external commissure may be divided with advantage; care being taken to carry the incision sufficiently deep to sever its fibrous attachments. The practice of cutting across, or excising enlarged vessels running upon the conjunctiva, is also wrong. These serve, by increasing its nutrition, to maintain the vitality and integrity of the cornea under the irritation to which it is constantly subjected, and gradually contract and disappear spontaneously when that is removed.

There is at present in the Hospital, a striking proof that amendment is sometimes possible under circumstances apparently desperate. A patient was admitted with the lids enormously thickened and otherwise diseased from the long continuance of chronic inflammation, their lining membrane disorganized, and the cornea covered to the depth of two or three lines with a fungous growth which completely obstructed vision. This was removed in the first instance by the scissors, and afterwards by the application in substance of the nitrate of silver; the lids were restored to their proper position by the excision of a portion of their integuments; and the irritation being thus removed, the process of clearing commenced, and the unhappy patient, still improving, has already recovered a very useful degree of vision.

Several cases of amaurosis presented themselves during the term, but in a brief commentary like the present, little can be said of a symptom depending upon so many morbid deviations, organic, functional, and sympathetic. It was connected in several instances, with derangement of the digestive system; but in others its etiology was more obscure. The affection arises more frequently, perhaps, than is generally supposed, from nervous exhaustion induced either by the excessive, or unnaturally prolonged activity of the generative organs.

Among the operations performed during the Quarter, amounting in the aggregate to seventy-five: eighteen were for cataract—eight lenticular and ten capsular or fibrinous;—seven for entropium; four, division of the external commissure; four, for artificial pupil, and one for fistula lachrymalis. Twenty-one persons applied for the removal of foreign bodies—generally a particle of iron—from the cornea; and in nine or ten cases of symblepharon, or adhesion of the lids to the globe, the connecting bands were divided, and various devices employed to prevent, or obviate the effects of, the contraction of the granulations. In a few instances, where the connection was mediate and of small extent, and the edges could be brought together by suture—the parts being also maintained, for a time, in a state of separation—something was gained thereby, and greater freedom of motion secured to the lid; but in general, so great is the tendency to the reproduction of the adhesions, little or no benefit follows such operations. Foreign bodies imbedded in the cornea, may commonly be removed without difficulty, by the edge of a cataract needle; but, if this cannot readily be done, and the particle is of small size, it is better to cover the eye with a wetted compress to restrain the motion of the lids and abate irritation; and to wait—contrary to the advice of some authorities—until ulceration has somewhat loosened the offending body, than to incur the risk of inflicting too much injury upon the organ. In one instance, a piece of metal entering the cornea obliquely, divided the iris near the external ciliary margin, leaving a small permanent aperture; which, however, did not interfere with vision.

The single case of fistula lachrymalis reported, was relieved by the introduction of a style. It is too much the practice with some individuals to operate in all conditions of this complaint; whereby much mischief is done, and a large amount of suffering unnecessarily inflicted. A spontaneous restoration of the passage under favoring circumstances is not impossible, and common sense, as well as sound pathology, inculcates the propriety of allowing time for vascular excitement to subside, and also for the correction of any constitutional aberration. In some instances where there is no fistulous opening, the inconvenience of the stillicidium is a lesser evil than would probably result from operative interference; and the advice given to several applicants, was in accordance with this opinion.

In two cases of operation for artificial pupil, where a small segment only of the cornea was transparent at its circumference, the corresponding portion of the iris was separated from its ciliary connections by a very fine forceps introduced through a small incision of the cornea near its centre; the detached portion, partially drawn out, was excised by the scissors, and a degree of vision restored which was highly valued and gratefully acknowledged by those who had long lived in darkness. In a case of closure of the pupil with fibrinous exudation from inflammation following a previous operation for cataract, an attempt was first made to break up the adhesions and enlarge the aperture by the needle introduced through the sclerotica. Failing in this, the cornea was opened near its circumference, and the iris divided across its centre by means of Maunoir's scissors. The fibres immediately retracted, and the patient—an old man of seventy-five years—recovered very useful vision. The lateral curvature of this instrument being rather an impediment than otherwise, a straight pair has been made, with blades shorter and of exceeding delicacy.

The eighteen operations for cataract, a few of which were repetitions, were all successful in the degree anticipated. In most of the capsular cases, the removal of the opaque body was accomplished by the introduction of the forceps above mentioned through a small section of the cornea; but in one or two instances, where the obstruction was caused by a thin, semi-transparent pellicle, this was effectually broken up by the needle introduced through the sclerotica. An opaque capsule, if it preserve its elasticity, may contract and disappear when divided, but it is never absorbed; and extraction through the cornea is, therefore, the only proper operation. Two of the patients, hopeless of relief, had been sent to the city with a view to admission into the Asylum for the Blind. In one of these, a girl of eleven years, the operation for congenital cataract instead of being performed in infancy, had unhappily been postponed until the child attained her ninth year, and the eyes had acquired in consequence an oscillatory motion. The operation was then performed in the country; the crystallines were removed by absorption; and the result was, an opaque capsule in the left eye, and a fibrinous cataract in the right. The one was removed by extraction through the cornea, and the other was broken up by the needle through the sclerotica.

The operation by solution is that most commonly performed at the Hospital for the removal of lenticular cataract; as, all circumstances considered, involving perhaps less risk to the organ than any other; care being taken to destroy the central portion of the capsule, and loosen or divide the substance of the lens with as little disturbance as possible of their connexions with surrounding parts. In one instance, in which the lens was manifestly hard and the vitreous humor disorganized, depression or couching was attempted; but the lens rising again behind the pupil, notwithstanding every effort to prevent it, the needle was withdrawn, and extraction performed by opening the cornea, and removing the opaque crystalline with the hook. In another case in which extraction was performed, the patient had long been blind from entropium. The lids of both eyes were inverted, the tarsal borders rounded from lippitudo, the cartilages incurvated, their lining membrane inflamed, thickened, and otherwise diseased; the corneæ obscured by vascularity and panniform opacity of their conjunctival covering, and both the iris and pupil totally invisible. The lids were restored to their natural position by the excision of a transverse portion of the integuments at their ciliary margin, and the constriction of the globe relieved by the division of the external commissures; but as the corneæ gradually regained their transparency, it unhappily became evident that a dense cataract occupied the pupil. From the appearance of the lens, hard and invested with an opaque and thickened capsule, and the small size and sunken condition of the eye, the operations of solution and depression were deemed inexpedient; and extraction was resolved upon, notwithstanding the presence of many of those circumstances which are generally thought to contra-indicate that mode of operation. No difficulty was experienced in its performance, the lens and capsule were both removed without accident, the edges of the wound gradually united; and though a reproduction to some extent of the external inflammation necessarily followed, this is again subsiding under the use of appropriate measures.

*Philadelphia, July*, 1854.

